# Proteasome inhibition and mechanism of resistance to a synthetic, library-based hexapeptide

**DOI:** 10.1007/s10637-018-0569-x

**Published:** 2018-02-14

**Authors:** Ruud Oerlemans, Celia R. Berkers, Yehuda G. Assaraf, George L. Scheffer, Godefridus J. Peters, Sue Ellen Verbrugge, Jacqueline Cloos, Jerry Slootstra, Rob H. Meloen, Robert H. Shoemaker, Ben A. C. Dijkmans, Rik J. Scheper, Huib Ovaa, Gerrit Jansen

**Affiliations:** 1 Departments of Rheumatology, Amsterdam Rheumatology and Immunology Center, Cancer Center Amsterdam, Rm 2.46, VU University Medical Center, De Boelelaan 1117, 1081 HV Amsterdam, The Netherlands; 2 Biomolecular Mass Spectrometry and Proteomics, Bijvoet Center for Biomolecular Research, Utrecht University, Utrecht, The Netherlands; 3 The Fred Wyszkowski Cancer Research Laboratory, Faculty of Biology, Technion-Israel Institute of Technology, Haifa, Israel; 4 Department of Pathology, VU University Medical Center, Cancer Center Amsterdam, Amsterdam, The Netherlands; 5 Department of Medical Oncology, VU University Medical Center, Cancer Center Amsterdam, Amsterdam, The Netherlands; 6 Department of Pediatric Oncology/Hematology, VU University Medical Center, Cancer Center Amsterdam, Amsterdam, The Netherlands; 7 Pepscan Therapeutics, Lelystad, The Netherlands; 8 Chemopreventive Agent Development Research Group, Division of Cancer Prevention, National Cancer Institute, National Institutes of Health, Bethesda, MD USA; 9 Division of Cell Biology II, Netherlands Cancer Institute, Amsterdam, The Netherlands; 10 Department of Chemical Immunology, Leiden University Medical Center, Leiden, The Netherlands

**Keywords:** Proteasome, Proteasome inhibitors, Bortezomib, Cytotoxic peptides, Drug resistance, ABC drug efflux transporters

## Abstract

*Background* The hexapeptide 4A6 (Ac-Thr(tBu)-His(Bzl)-Thr(Bzl)-Nle-Glu(OtBu)-Gly-Bza) was isolated from a peptide library constructed to identify peptide-based transport inhibitors of multidrug resistance (MDR) efflux pumps including P-glycoprotein and Multidrug Resistance-associated Protein 1. 4A6 proved to be a substrate but not an inhibitor of these MDR efflux transporters. In fact, 4A6 and related peptides displayed potent cytotoxic activity via an unknown mechanism. *Objective* To decipher the mode of cytotoxic activity of 4A6. *Methods* Screening of 4A6 activity was performed against the NCI60 panel of cancer cell lines. Possible interactions of 4A6 with the 26S proteasome were assessed via proteasome activity and affinity labeling, and cell growth inhibition studies with leukemic cells resistant to the proteasome inhibitor bortezomib (BTZ). *Results* The NCI60 panel COMPARE analysis revealed that 4A6 had an activity profile overlapping with BTZ. Consistently, 4A6 proved to be a selective and reversible inhibitor of β5 subunit (PSMB5)-associated chymotrypsin-like activity of the 26S proteasome. This conclusion is supported by several lines of evidence: (i) inhibition of chymotrypsin-like proteasome activity by 4A6 and related peptides correlated with their cell growth inhibition potencies; (ii) 4A6 reversibly inhibited functional β5 active site labeling with the affinity probe BodipyFL-Ahx_3_L_3_VS; and (iii) human myeloid THP1 cells with acquired BTZ resistance due to mutated *PSMB5* were highly (up to 287-fold) cross-resistant to 4A6 and its related peptides. *Conclusion* 4A6 is a novel specific inhibitor of the β5 subunit-associated chymotrypsin-like proteasome activity. Further exploration of 4A6 as a lead compound for development as a novel proteasome-targeted drug is warranted.

## Introduction

The central role that the ubiquitin-proteasome system plays in intracellular protein degradation has been exploited as a potential therapeutic target for the treatment of hematological malignancies, solid tumors and cancer and chronic inflammatory diseases [1–10]. The 26S–proteasome complex is made up of a 20S core unit, consisting of 4 stacked heptameric rings, which form an α_7_β_7_β_7_α_7_ complex, and is capped by two 19S regulatory units [11–13]. The 20S core unit harbors the proteasome’s catalytic domain which is responsible for caspase-like, trypsin-like and chymotrypsin-like activities, associated with the β1, β2 and β5 subunit, respectively [14]. Several types of proteasome inhibitors have been described that reversibly or irreversibly inhibit proteasome activity by targeting one or more of these β subunits [15–19]. Bortezomib (Velcade®, PS341) was the first proteasome inhibitor that was clinically approved and registered for the treatment of refractory multiple myeloma [2, 20].

Bortezomib (BTZ) is a potent reversible proteasome inhibitor (IC_50_: 3–5 nM) that primarily targets the β5 subunit of the proteasome, although the β1 subunit and its immunoproteasome counterparts are also targeted [15, 21]. While bortezomib is clinically well tolerated, prolonged administration may result in neurotoxicity and drug resistance may emerge [15, 22–24]. Thus, alternative proteasome inhibitors are in demand [7, 17, 25–31].

**Fig. 1:**
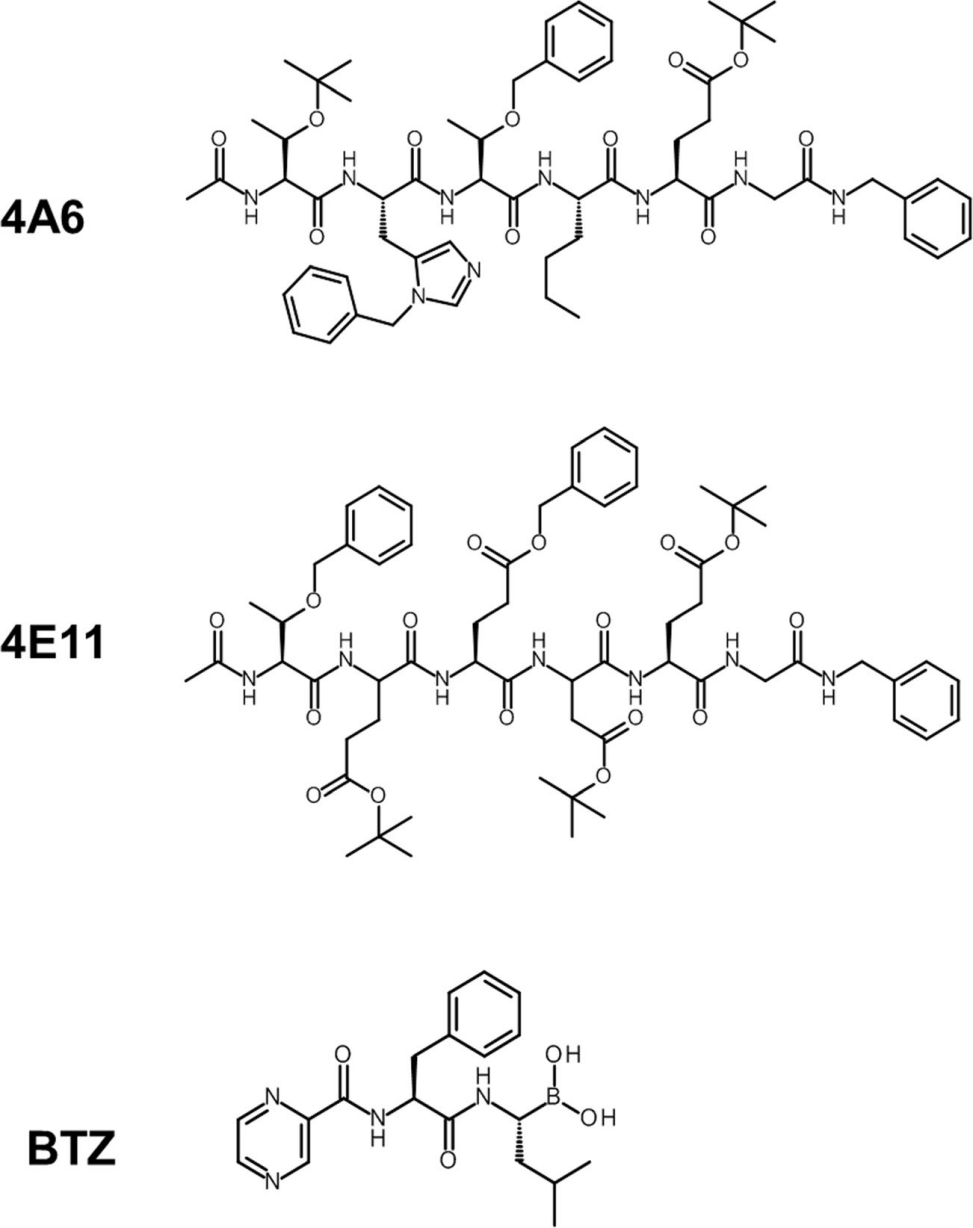
*Chemical structures of 4A6, 4E11 and bortezomib*

The hexapeptide 4A6 (Ac-Thr(tBu)-His(Bzl)-Thr(Bzl)-Nle-Glu(OtBu)-Gly-Bza) [32] (Fig. 1) was identified from a peptide library constructed to identify peptide-based inhibitors of multidrug resistance (MDR) efflux transporters including P-glycoprotein (Pgp/ABCB1) and Multidrug Resistance-associated Protein 1 (MRP1/ABCC1) [33]. These ATP-binding cassette transorters extrude a plethora of structurally and mechanistically distinct cytotoxic agents and thus confer multidrug resistance upon various cancer cells [34–36]. In recent years, several types of peptides (linear/cyclic, neutral/ hydrophobic) have been identified for their interaction with MDR efflux transporters and/or their potential chemosensitizing capacity; these include cyclosporin A, gramicidin D, valinomycin, ALLN, dolastatin 10, pepstatin A, leupeptin and reversin 121 [37–45]. Likewise, 4A6 was found to be a substrate of the MDR efflux transporters ABCB1 and ABCC1, but lacked the ability to reverse efflux pump MDR [32]. In fact, 4A6 and related peptides displayed potent cytotoxic effects via an unknown mechanism [32]. Here we uncovered the mode of action of 4A6 and provide ample evidence that it exerts its pharmacological activity by blocking the chymotrypsin-like activity of the proteasome. This finding warrants the exploration of 4A6 as a lead compound for further development as a novel proteasome-targeted drug.

## Materials and methods

### Reagents

Bortezomib (Pyrazylcarbonyl-Phe-Leu-boronate) was provided by the VUmc Hospital Pharmacy Department. The cytotoxic peptides 4A6 (Ac-Thr(tBu)-His(Bzl)-Thr(Bzl)-Nle-Glu(OtBu)-Gly-Bza) (monomer and dimer form) and 4E11 (Ac-Thr(OBzl)-Glu(OtBu)-Glu(OBzl)-Asp(OtBu)-Glu(OtBu)-Gly-Bza) were synthesized as described previously [32]. P121/Reversin (Boc-Asp(OBzl)-Lys-(Z)-OtBu) was kindly provided by Prof. Dr. B. Sarkadi (Budapest, Hungary).

Protease Inhibitor Cocktail (PIC) was obtained from Boehringer Mannheim (Ingelheim, Germany). RPMI-1640 medium and fetal calf serum were obtained from Gibco Chem. Co (Grand Isl., NY, U.S.A). All fluorogenic substrates (Suc-Leu-Leu-Val-Tyr-amc, Ac-Arg-Leu-Arg-amc and Z-Leu-Leu-Glu-amc), the proteasome inhibitors Ac-APnLD-H and leupeptin, and all proteasome subunit-related antibodies (β1, β2, and β5) were purchased from Biomol (Plymouth Meeting, PA, U.S.A.). Anti-ubiquitin antibody (sc-8017) was purchased from Santa Cruz (USA). Ruthenium Red was obtained from Sigma Chem Co (USA).

### Synthesis of ac-Thr(tBu)-his(Bzl)-Thr(Bzl)-Nle-OH

The tetramer (Ac-Thr(tBu)-His(Bzl)-Thr(Bzl)-Nle-OH), the major cleavage product of 4A6, was synthesized by standard Fmoc-based solid phase peptide synthesis on FmocNorLeu Sasrin resin. The FmocNorLeu resin was prepared by esterification of FmocNorLeu-OH (10 equivalents) with the unloaded resin using *N*,*N*′-diisopropylcarbodiimide (DIC, 10 equivalents) in dimethylformamide. The resin was deprotected with 1% trifluoroacetic acid (TFA) in methylenechloride for 2.5 h followed by precipitation of the peptide with diethylether and HPLC purification (Waters 1525 EF HPLC system).

### Cell cultures

Human monocytic/macrophage THP1 cells (ATTC, Manassas, USA) were cultured in RPMI-1640 medium supplemented with 5% fetal calf serum, 20 mM HEPES, 2 mM glutamine and 100 μg/ml penicillin/ streptomycin at 5% CO_2_ and 37 °C. Cell cultures were seeded at a density of 3 × 10^5^ cells/ml and refreshed twice weekly. Bortezomib (BTZ)-resistant THP1 cell lines were obtained by stepwise increasing extracellular concentrations of BTZ over a period of 6 months [46]. In this study, BTZ-resistant THP1 variants were used and grown in the presence of 50 nM (THP1/BTZ_50_), 100 nM (THP1/BTZ_100_) and 200 nM bortezomib (THP1/BTZ_200_) (see Table 1). Some specific experiments also included THP1/BTZ_100_ cells that were cultured in the absence of BTZ for 6 months (further designated as THP1/BTZ _(−100)_ cells). Mouse thymoma EL4 and human multiple myeloma H929 cells were cultured in RPMI-1640 medium supplemented with 8% fetal calf serum and 100 μg/ml penicillin/streptomycin at 5% CO_2_ and 37 °C.

**Table 1:** Growth inhibitory effects of cytotoxic peptides on THP1 and bortezomib-resistant THP1 cells

Drug	IC_50_ (μM)	[Resistance Factor]		
	THP1/WT	THP1/BTZ_50_	THP1/BTZ_100_	THP1/BTZ_200_
4A6	0.26 ± 0.06 [1]	44	117	287
4A6-dimer	0.80 ± 0.09 [1]	>63*	>63*	>63*
4E11	3.9 ± 0.8 [1]	>13*	>13*	>13*
CsA	3.8 ± 1.0 [1]	0.9	0.8	ND
Bortezomib^#^	0.0033 ± 0.0006 [1]	45	79	129

Results depicted are the mean of at least 3 separate experiments ± S.D.

*ND* Not determined, *CsA* cyclosporin A

^#^Data from Oerlemans et al. [46]

*Solubility of peptide in medium is limited to a concentration of 50 μM

### Screening of 4A6 using the NCI60 tumor cell line panel

The NCI 60 human tumor cell line screen was used to assess the activity profile of 4A6 against a panel of tumor cell lines of various cell lineage [47]. Concentrations of 4A6 eliciting 50% growth inhibition (GI50) were determined after 48 h drug exposure. 4A6 sensitivity for each individual cell line is depicted relative to the mean GI50 of the total cell line panel.

### 4A6 cleavage assay

Proteasome was purified from bovine liver as described previously [48]. For digestion assays, 1 μg proteasome was incubated with 1 μg 4A6 in 50 μl of 50 mM Tris-HCl buffer pH 8.5 at 45 °C for 16 h. Subsequently, the reaction mixture was lyophilized and peptides purified using reversed-phase ZipTip®_C18_ tips (Millipore). The purified peptide mixture was mixed in a 1:1 ratio with 10 mg/ml 2,5-dihydroxybenzoic acid (DHB, Bruker Daltonik) matrix solution in 0.1% TFA and spotted onto a MALDI (matrix assisted laser desorption/ ionization) target plate. MALDI-TOF analysis was performed on an Autoflex, linear MALDI-TOF-MS (Bruker Daltonik GmbH, Bremen, Germany). Spectra were analyzed with flexAnalysis software (Bruker Daltonik).

### Growth inhibition assays

Evaluation of drug sensitivity was carried out as described before [49]. Cells were seeded at an initial density of 1.25 × 10^5^ cells/ml in individual wells of a 24-well plate containing up to 50 μl of drug solutions. Inhibition of cell growth was determined after 72 h of incubation at 37 °C by determining the number of viable cells viable cells using trypan blue exclusion. The drug concentration required to inhibit cell growth by 50% compared to untreated controls was defined as the IC_50_.

### Western blot analysis (ubiquitinated proteins/proteasome subunits)

Western blot analysis to determine protein levels of (i) β1, β2 and β5 proteasome subunits and (ii) the accumulation of ubiquitinated proteins after treatment with 4A6 was performed essentially as described previously [46, 49]. Cells were harvested in the mid-log phase of growth and washed 3 times with ice-cold buffered saline pH 7.4. Total cell lysates of 5 × 10^6^ cells were prepared by resuspension in 500 μl lysis buffer containing: 50 mM Tris-HCl (pH 7.6), 5 mM dithiotreitol, 20 μl PIC (Protease Inhibitor Cocktail; 1 tablet/ml H_2_O), 20% glycerol and 0.5% NP-40. The suspension was sonicated (MSE sonicator, amplitude 7, for 3 × 5 s with 20 s time intervals at 4 °C) and centrifuged in an Eppendorf micro centrifuge (5 min, 12,000 rpm, 4 °C). Protein content of the supernatant was determined by the Bio-Rad protein assay. 20–30 μg of total cell lysates were fractionated on a 10% polyacrylamide gel containing SDS and transferred onto a PVDF membrane. The membranes were pre-incubated overnight at 4 °C in blocking buffer (5% Bio-Rad Blocker in TBS-T; 10 mM Tris-HCl, pH 8.0, 0.15 M NaCl, 0.1% Tween-20) to prevent non-specific antibody binding. After blocking, the membranes were incubated for 1 h at room temperature with primary antibodies for proteasome subunit β1 (1:1000, PW8140), β2 (1:1000, PW8145) and β5 (1:1000, PW8895) or ubiquitin (1:1000, Santa-Cruz, SC-8017). An antibody to α-tubulin was used (1:1000, Santa Cruz, sc-8035) to check and normalize for any loading differences. After 3 washing steps with TBS-T, the membranes were incubated for 1 h with HRP-labelled donkey-anti-rabbit (1:6000, Amersham, UK) or goat-anti-mouse (1:6000, Dako, Glostrup, Denmark) as secondary antibody. Detection of antibody binding was followed by chemoluminescence using Supersignal (Pierce Biotechnology, Rockford, USA) according to the manufacturers’ instructions. Digital Image acquisition was performed using the Versadoc Imaging System (Biorad Lab., Veenendaal, The Netherlands). The signal intensity was determined densitometrically using Quantity One software (Bio-Rad) and was expressed relative to the intensity of the α-tubulin signal.

### Proteasome activity in cell lysates and intact cells

Chymotrypsin-like, trypsin-like and caspase-like proteolytic activities of the proteasome were determined in freshly prepared cell lysates as described previously [21, 46]. Five million untreated or bortezomib-exposed THP1 cells were washed 3 times with ice-cold PBS and pelleted by centrifugation (5 min, 12,000 RPM, 4 °C). Cell pellets were then resuspended in an ATP-containing lysis buffer; 10 mM Tris-HCl buffer (pH 7.8) containing 5 mM ATP, 0.5 mM DTT and 5 mM MgCl_2_, and kept on ice for 10 min. For complete lysis, cells were sonicated (MSE sonicator, amplitude 7, for 3 × 5 s with 20 s time intervals at 4 °C) followed by centrifugation (5 min, 12,000 RPM, 4 °C) to remove cell debris. The supernatant was collected and protein concentration was determined using the Bio-Rad protein assay. Fluorogenic substrates to measure the chymotrypsin-like, trypsin-like and caspase-like activity were Suc-Leu-Leu-Val-Tyr-amc, Ac-Arg-Leu-Arg-amc and Z-Leu-Leu-Glu-amc, respectively, all at a final concentrations of 100 μM. The substrates were incubated with 20 μg of total cell protein extract in the presence or absence of specific inhibitors (bortezomib for chymotrypsin-like activity, Ac-APnLD-H for caspase-like activity and leupeptin- for trypsin-like activity) in a total assay volume of 200 μl. The release of amc (7-amino-4-methyl-coumarin) was monitored online over a 2-h time period at 37 °C with 5 min intervals. Fluorescence was measured on a Tecan SpectraFluor apparatus (Giessen, The Netherlands) using excitation and emission wavelengths of 360 and 465 nm, respectively. Proteolytic activity was calculated from the slopes of the linear portion of the curves. All results were expressed as percentage relative to untreated THP1/WT cells (100%). Inhibition of chymotrypsin-like activity in intact cells was measured by the Proteasome-Glo™ cell-based assay (Promega, Leiden, The Netherlands), using Suc-LLVY-aminoluciferin as a substrate, according to the manufacturer’s instructions.

### Proteasome affinity labelling

Proteasome activity profiling assays were performed as described [50, 51] using a close analog of the BodipyFL probe. Briefly, mouse EL4 thymoma cells were incubated at 37 °C for 2 or 24 h with increasing concentrations of 4A6, followed by a 1 h chase with 500 nM probe. In other experiments, human H929 myeloma cells were incubated at 37 °C with 1 μM 4A6 (2 h), 5 μM MG132 (1 h) or 20 nM bortezomib (1 h) and subsequently probed with 500 nM probe (1 h), either directly or after a washing and recovery step. Cells were harvested and lysed for 30 min in NP40 lysis buffer (50 mM Tris, pH 7.4, 150 mM NaCl, 1% NP40) at 4°C. The Bradford assay was used to measure protein content. Proteins were denatured by boiling in reducing sample buffer and analyzed by 12% SDS-PAGE using NuPAGE pre-cast gels (Invitrogen). Gels were then scanned for fluorescence emission using a ProXPRESS 2D Proteomic imaging system (Perkin Elmer). Images were analyzed using Totallab analysis software (Nonlinear Dynamics, Newcastle upon Tyne, UK). Sypro staining served as a loading control.

#### Apoptosis assay

Induction of apoptosis was analyzed by flow cytometry using APOPTEST™-FITC A700 (VPS Diagnostics, Hoeven, the Netherlands) according to the instructions of the manufacturer. In short, induction of apoptosis was determined after 24 h’ drug exposure. One million cells were harvested and washed 3 times with ice-cold PBS. The cell pellet was incubated for 30 min with 7-Amino-actinomycin D (7-AAD) on ice followed by incubation with Annexin-V according to the instructions of the manufacturer. Annexin-V (early apoptosis) and 7-AAD (late apoptosis) staining was measured by flow cytometry (Beckton & Dickinson, FACScalibur) and analysed using FCSexpress V3 software (Denovo software, Thornhill, Canada).

#### Statistics

Statistical analysis was performed using Analysis of Variance between groups (ANOVA) in Graphpad prism version 6.0. *P* values <0.05 were considered to be statistically significant.

## Results

### 4A6 vs bortezomib activity against NCI60 panel of tumor cell lines

**Fig. 2:**
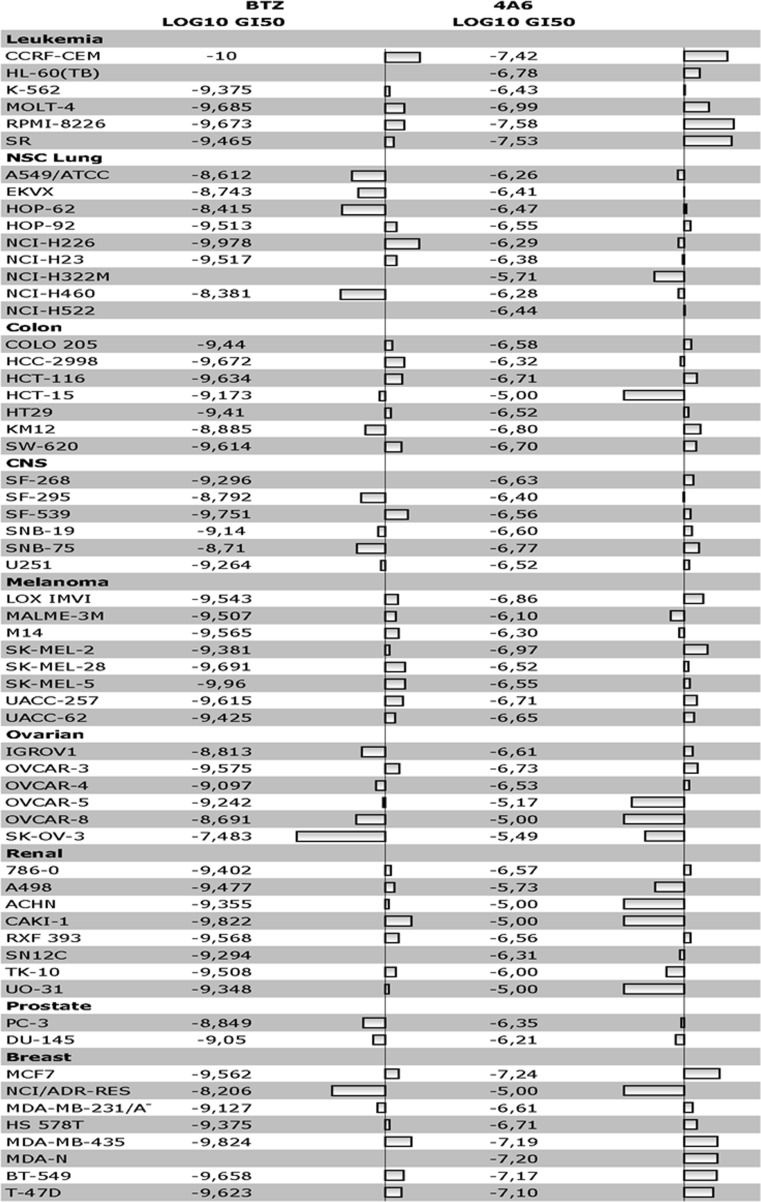
*Cytotoxic activity profiles of bortezomib* vs *4A6 against the NCI-panel of 60 malignant cell lines.* Data are based on 48 h’ drug exposure and presented as log GI_50_ for each individual tumor cell line and as GI_50_ relative to the mean GI_50_ of all cell lines tested

In order to get an initial insight regarding the cytotoxic activity of 4A6, we first tested 4A6 in the NCI60 tumor cell line panel that is composed of 60 malignant cell lines of distinct tissue lineage [47]. 4A6 showed remarkable activity towards a panel of leukemia, breast cancer, melanoma, and to some extent colon cancer cells (Fig. 2). In contrast, 4A6 proved rather inactive towards a panel of renal cancer cells and lung cancer cells. Moreover, cells with high levels expression of the multidrug efflux transporter Pgp, including HCT-15, ACHN, UO-31 and NCI/ADR-RES [52], displayed marked resistance to 4A6. COMPARE analysis of GI_50_ values for 4A6 in the NCI-panel of 60 cell lines showed a correlation coefficient (r) of 0.37 with bortezomib (BTZ), an established proteasome inhibitor drug. A side by side comparison of the activity profile of 4A6 and BTZ in the NCI60 panel of tumor cell lines showed overlapping sensitivities (Fig. 2), albeit based on mean log_10_GI_50_ concentrations obtained after 2 days of drug exposure, BTZ was 2–3 orders of magnitude more potent than 4A6. These results demonstrate that 4A6 has an overlapping activity profile with BTZ against the NCI60 panel of tumor cell lines; however, in contrast to BTZ, 4A6 activity was compromised by the presence of a Pgp-dependent MDR phenotype.

### Cells with acquired resistance to peptide-based proteasome inhibitor bortezomib are cross-resistant to the cytotoxic peptides 4A6 and 4E11

**Fig. 3:**
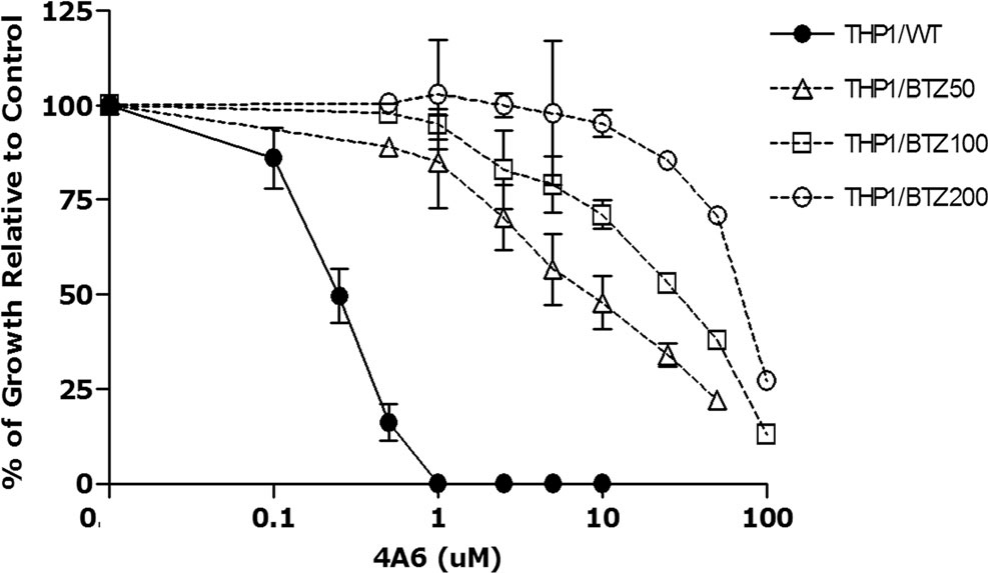
*Cross-resistance to 4A6 in bortezomib-resistant cells.* Dose response curve for 4A6-induced growth inhibition of wild type (WT) human myelomonocytic THP1 cells and proteasome (bortezomib, BTZ)-resistance selected variants; THP1/BTZ_50_, THP1/BTZ_100_ and THP1/BTZ_200_, selected for growth in extracellular concentrations of 50 nM, 100 nM and 200 nM BTZ, respectively. Results depicted are the means of 3 experiments ± S.D. 4A6 exposure time: 72 h

Because of the overlapping activity profile of 4A6 and BTZ in the NCI60 panel, we tested 4A6 in human THP1 cell lines with acquired resistance to BTZ. These cell lines displayed cross-resistance to other known peptide-based proteasome inhibitors (e.g. ALLN, MG132), but also to the linear cytotoxic hexapeptide 4A6, the latter of which has an unknown mechanism of action [46]. To further explore the molecular basis of this observation, THP1 cells with various levels of BTZ-resistance were screened for their sensitivity to 4A6, a dimer form of 4A6, another linear cytotoxic hexapeptide 4E11 (Fig. 1), and the cyclic cytotoxic decapeptide cyclosporin A (Table 1). Within this panel of cytotoxic peptides, 4A6 was the most potent inhibitor of THP1 cell growth (IC_50_: 0.26 μM), followed by a 3-fold lower potency for the 4A6 dimer and a 15-fold lower potency for 4E11 and cyclosporin A (Table 1). These bortezomib-resistant cell lines displayed the highest levels (up to 287-fold) of cross-resistance to 4A6 (Table 1, Fig. 3) and >60-fold cross resistance to the 4A6-dimer. With respect to the peptide 4E11, a consistently higher IC50 value compared to 4A6 (Table 1) along with limitations in solubility of peptides above a concentration of 50 μM, allowed for the assessment of relatively low level (>13-fold) cross-resistance to 4E11. No cross-resistance of bortezomib-resistant cells was observed for cyclosporin A. Collectively, these results indicate that the peptides 4A6 and 4E11 share properties with known inhibitors of the ubiquitin-proteasome system, including BTZ.

### 4A6 is a potent inhibitor of chymotrypsin-like proteasome activity

**Fig. 4:**
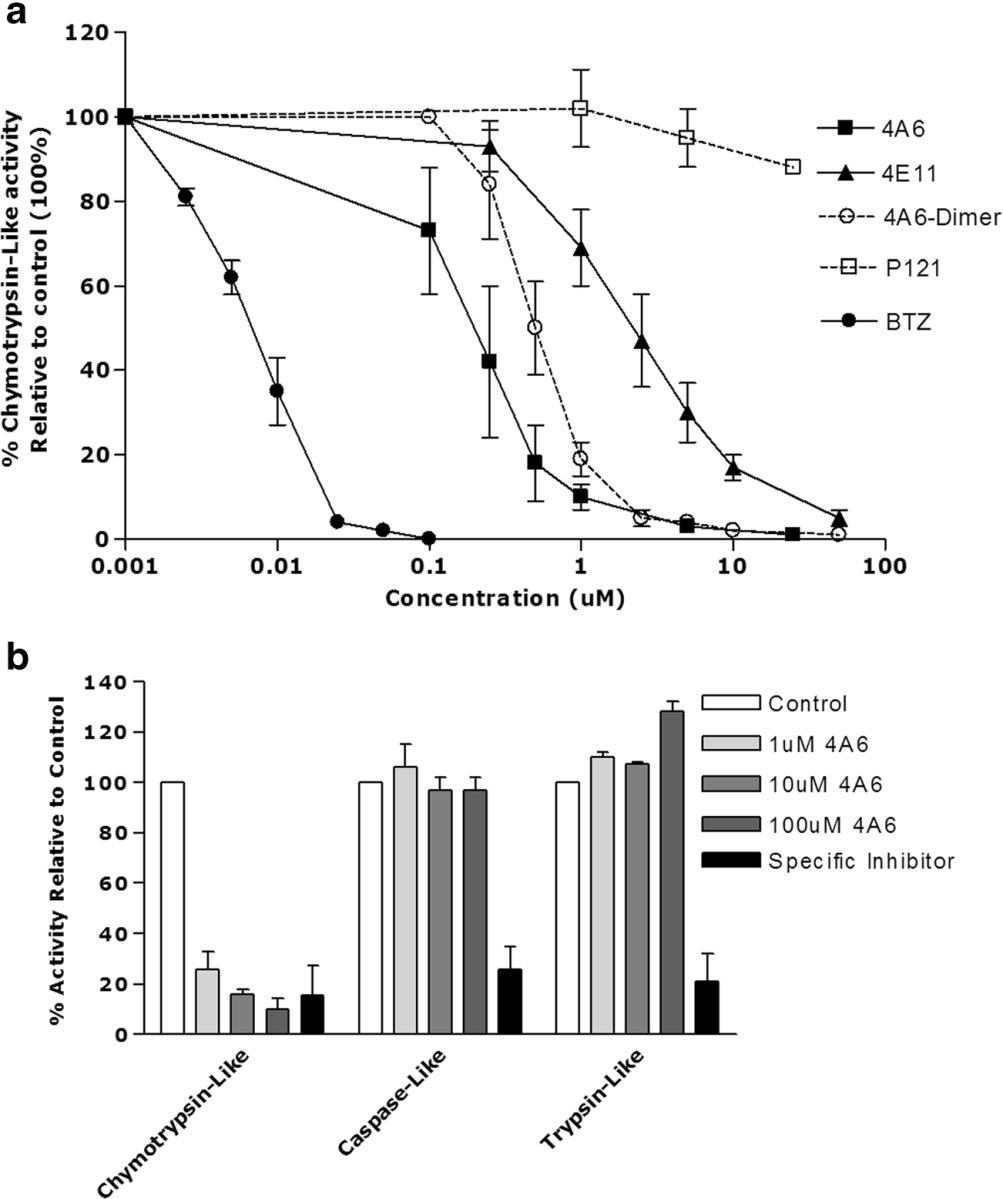
**a** *Potent inhibition of proteasome activity by the hexameric 4A6 peptide.* Luminescent cell-based proteasome assay measuring inhibition of chymotrypsin-like proteasome activity in intact THP1 cells after 1 h exposure to BTZ, the hexameric peptide 4A6, 4A6-dimer, the hexameric peptide 4E11 and, as control, the tripeptide P121/Reversin, a peptide-based transport inhibitor of the MDR protein P-glycoprotein. Results represent the mean of 3 experiments ± S.D. **b** *Inhibition of chymotrypsin-like but not caspase-like and trypsin-like proteasomal activity by 4A6*. Chymotrypsin-like, caspase-like and trypsin-like proteasomal activities were determined with specific fluorogenic peptide substrates in cell extracts of THP1 cells after 1 h exposure to the indicated concentrations of 4A6. Controls for selective inhibition of chymotrypsin-like, caspase-like and trypsin-like activity included BTZ (10 nM), Ac-APnLP (25 μM) and leupeptin (20 μM), respectively. Results represent the mean of 3 separate experiments ± S.D

An intact cell-based luminogenic assay that monitors chymotrypsin-like proteasome activity was used to investigate whether the cytotoxic peptides 4A6 and 4E11 could exert their cytotoxic effect via inhibition of proteasome activity (Fig. 4). Indeed, 4A6 displayed a marked inhibition of chymotrypsin-like proteasome activity (IC_50_: 0.21 ± 0.05 μM) with a potency 28-fold lower than BTZ (IC_50_: 0.0074 ± 0.002 μM (Fig. 4a). Likewise, the 4A6-dimer and 4E11 were found to inhibit chymotrypsin-like proteasome activity, though with a lower potency than 4A6 (IC_50_: 0.49 ± 0.12 μM and 2.4 ± 0.5 μM, respectively). A control peptide Reversin 121, a transport inhibitor of the MDR efflux transporter P-gp [38], had no effect on proteasome activity (IC_50_: > > 25 μM). Hence, the potency ranking of proteasome inhibitory activity (bortezomib >4A6 > 4A6-dimer >4E11) tightly correlated with their capacity to inhibit cell growth of THP1/WT cells (Table 1).

To address whether or not 4A6 is also capable of inhibiting one or both of the other protease activities harbored by the proteasome, chymotrypsin-, caspase- and trypsin-like activities were measured in THP1 cell extracts in the absence or presence of 4A6. Consistent with results shown in Fig. 4a, 4A6 elicited potent inhibitory effects (84–93%) on chymotrypsin-like proteasome activity, but had no inhibitory effect on caspase- and trypsin-like activity over a wide concentration range of 1–100 μM (Fig. 4b). These results demonstrate that 4A6 is a potent and selective inhibitor of chymotrypsin-like proteasome activity.

### 4A6 is a reversible inhibitor of chymotrypsin-like proteasome activity

**Fig. 5:**
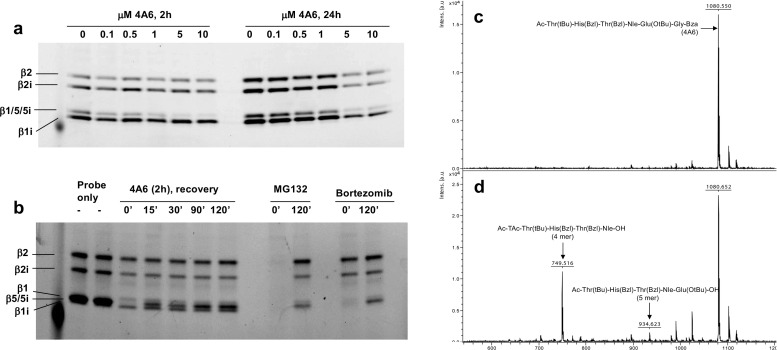
*4A6 is a reversible inhibitor of proteasome subunit β5*. **a** EL4 cells were incubated with the indicated concentrations of 4A6 for 2 or 24 h and then probed with a proteasome affinity probe as described in the Materials & Methods section. **b** H929 cells were incubated with 1 μM 4A6 (2 h), 20 nM BTZ (1 h) or 5 μM MG132 (1 h). Subsequently, cells were either probed directly with the affinity probe (0′) or resuspended in fresh medium without inhibitor and left to recover for the indicated times. As a control, non-treated cells were probed directly. A representative of 2 separate experiments is shown. **c** MALDI spectrum of 4A6 (m/z 1080.6). **d** MALDI spectrum of 4A6 after proteasomal digestion, showing the appearance one major cleavage product at m/z 749.5, which corresponds to Ac-Thr(tBu)-His(Bzl)-Thr(Bzl)-Nle-OH

Activity probing of constitutive and immunoproteasome β-subunits in EL4 cells that were pre-exposed to 4A6 for 2–24 h revealed a marked and specific inhibition of the β5 subunit with half maximal inhibition at 4A6 concentrations between 0.1 and 0.5 μM and complete inhibition at concentrations >5 μM (Fig. 5a). We next assessed whether this inhibition of β5-subunit could be recovered after removal of 4A6. Data shown in Fig. 5b illustrate that 4A6 is a reversible inhibitor of β5-subunit activity as initial recovery of activity could be observed already after 15 min of 4A6 drug removal and almost complete recovery after 2 h of 4A6 withdrawal. For comparison, the proteasome inhibitor MG132 blocked activity probing of all β-subunits, with a recovery 2 h after drug withdrawal (Fig. 5b). BTZ predominantly inhibited β5-subunit probing but affinity labeling was fully recovered within 2 h after drug withdrawal (Fig. 5b). We finally explored whether 4A6 remained intact as a peptide or could be subject to proteolytic cleavage when exposed to purified proteasomes. Comparison of mass spectra of the intact peptide (Fig. 5c) and the peptide after proteasomal digestion (Fig. 5d) showed that next to 4A6 (m/z 1080.6), one main additional peak appeared after digestion at m/z 749.5, corresponding to the 4-mer peptide Ac-Thr(tBu)-His(Bzl)-Thr(Bzl)-Nle-OH. A smaller peak appeared at m/z 934.6, corresponding to 5-mer peptide Ac-Thr(tBu)-His(Bzl)-Thr(Bzl)-Nle-Glu(OtBu)-OH. This indicates that 4A6 is predominantly cleaved at the P4-P5 position and to a lesser extent at the P5-P6 position. The main 4A6 proteasomal cleavage product, Ac-Thr(tBu)-His(Bzl)-Thr(Bzl)-Nle-OH was synthesized, but did not show any proteasome inhibitory effect or cell growth inhibitory potential (data not shown). Hence, these results suggest that 4A6 is a dual substrate and reversible inhibitor of proteasome subunit β5.

### Cellular exposure to 4A6 induces accumulation of ubiquitinated proteins and apoptosis but displays properties distinct of bortezomib

**Fig. 6:**
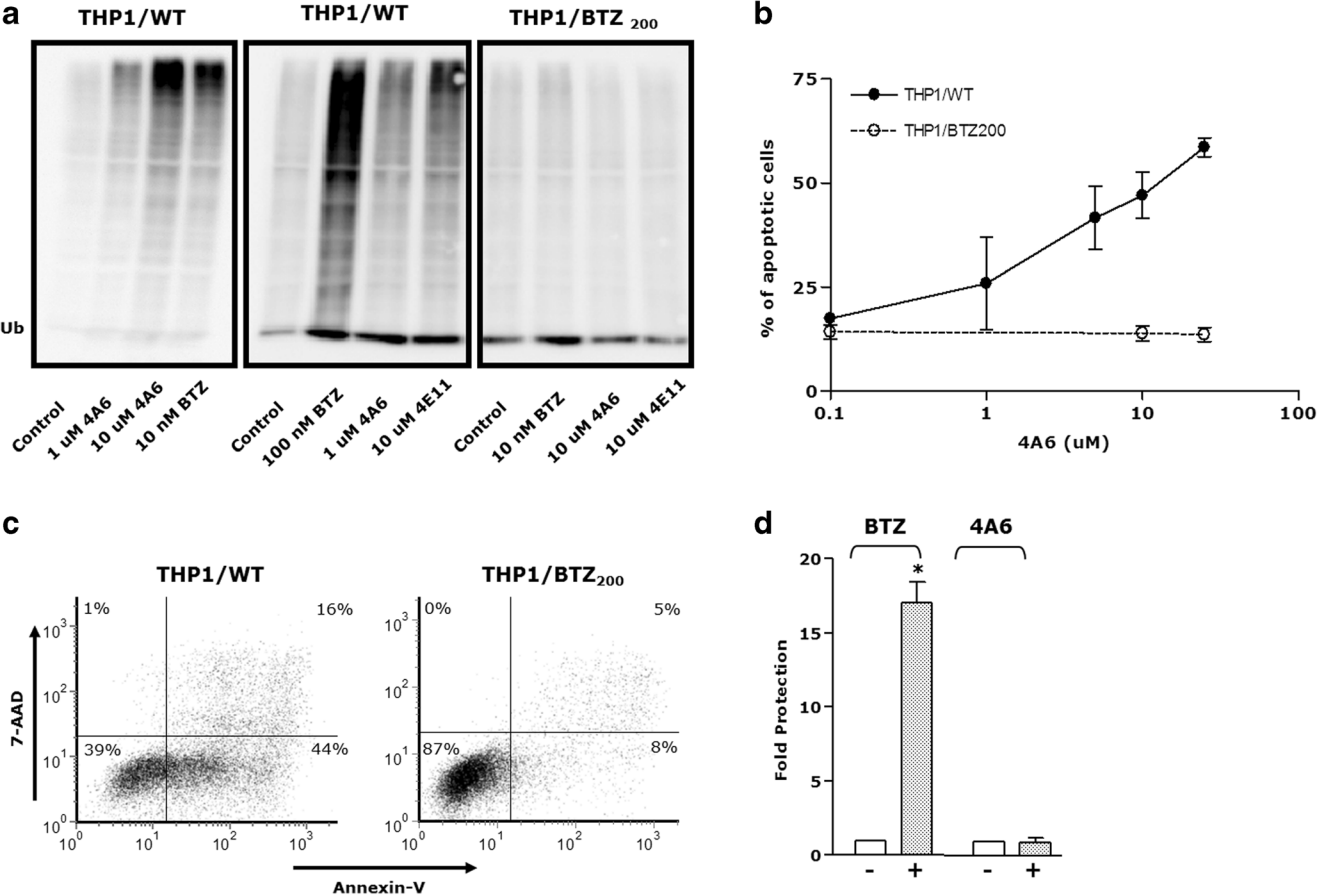
*4A6-induced accumulation of ubiquitinated proteins and induction of apoptosis in THP1/WT cells but not in BTZ-resistant cells.* **a** Accumulation of ubiquitinated proteins in THP1/WT cells and BTZ-resistant THP1/BTZ_200_ cells after 24 h’ exposure to the indicated concentrations of BTZ and 4A6. THP1/BTZ_200_ cells were allowed a 4 day drug washout period (control) before exposure to BTZ or 4A6. **b** Induction of apoptosis (Annexin-V positive cells) in THP1/WT cells and THP1/BTZ_200_ cells after 24 h’ exposure to a concentration range of 4A6. **c** A representative flow cytometric tracing of apoptosis induction (Annexin-V/7-AAD staining) following 24 h incubation of THP1/WT cells and THP1/BTZ_200_ cells with 25 μM 4A6. **d** *Ruthenium Red protects from BTZ-induced but not from 4A6-induced cell growth inhibition.* THP1 cells were incubated for 72 h with a concentration range of BTZ or 4A6 in the absence (−) or presence (+) of 25 μM Ruthenium Red. The ratio of IC_50_ values for BTZ and 4A6 in the presence or absence of Ruthenium Red is depicted as fold protection. Mean of 3 separate experiments ± S.D

One hallmark of proteasome inhibition is the accumulation of ubiquitinated proteins, which are toxic to cells and induce apoptosis [53, 54]. Exposure of THP1/WT cells to 4A6 and 4E11 for 24 h resulted, just as for the known proteasome inhibitor BTZ, in a marked accumulation of ubiquitinated proteins, illustrated by a characteristic smear upon Western blot probed with an anti-ubiquitin antibody (Fig. 6a). In contrast, the same concentrations of 4A6 and 4E11 did not provoke any accumulation of ubiquitinated proteins in bortezomib-resistant cells. Consistent with these observations was the efficient induction of apoptosis by 4A6 in parental THP1/WT cells but none by 4A6 (over a concentration range of 0–25 μM) in THP1/BTZ_200_ cells (Fig. 6b and c). For comparison, the anti-cancer drug and topoisomerase II inhibitor etoposide (VP16) was equally effective in inducing apoptosis in THP1/WT and THP1/BTZ_200_ cells (not shown).

To explore whether 4A6 shares properties with the known proteasome inhibitor BTZ, we investigated the ability of 4A6 to mimic a reported feature of BTZ, the disregulation of intracellular calcium homeostasis that triggers caspase activation and apoptosis [55]. This process could be counteracted by inhibitors of the mitochondrial calcium uniporter (e.g. Ruthenium Red), thereby providing a protective effect against BTZ [55]. While a marked abrogation of BTZ activity could be obtained by Ruthenium Red, no effect of this compound was observed with respect to 4A6 activity (Fig. 6d). These results suggest that 4A6 has no apparent impact on mitochondrial calcium homeostasis.

### 4A6 provokes proteasome β5 subunit induction

**Fig. 7:**
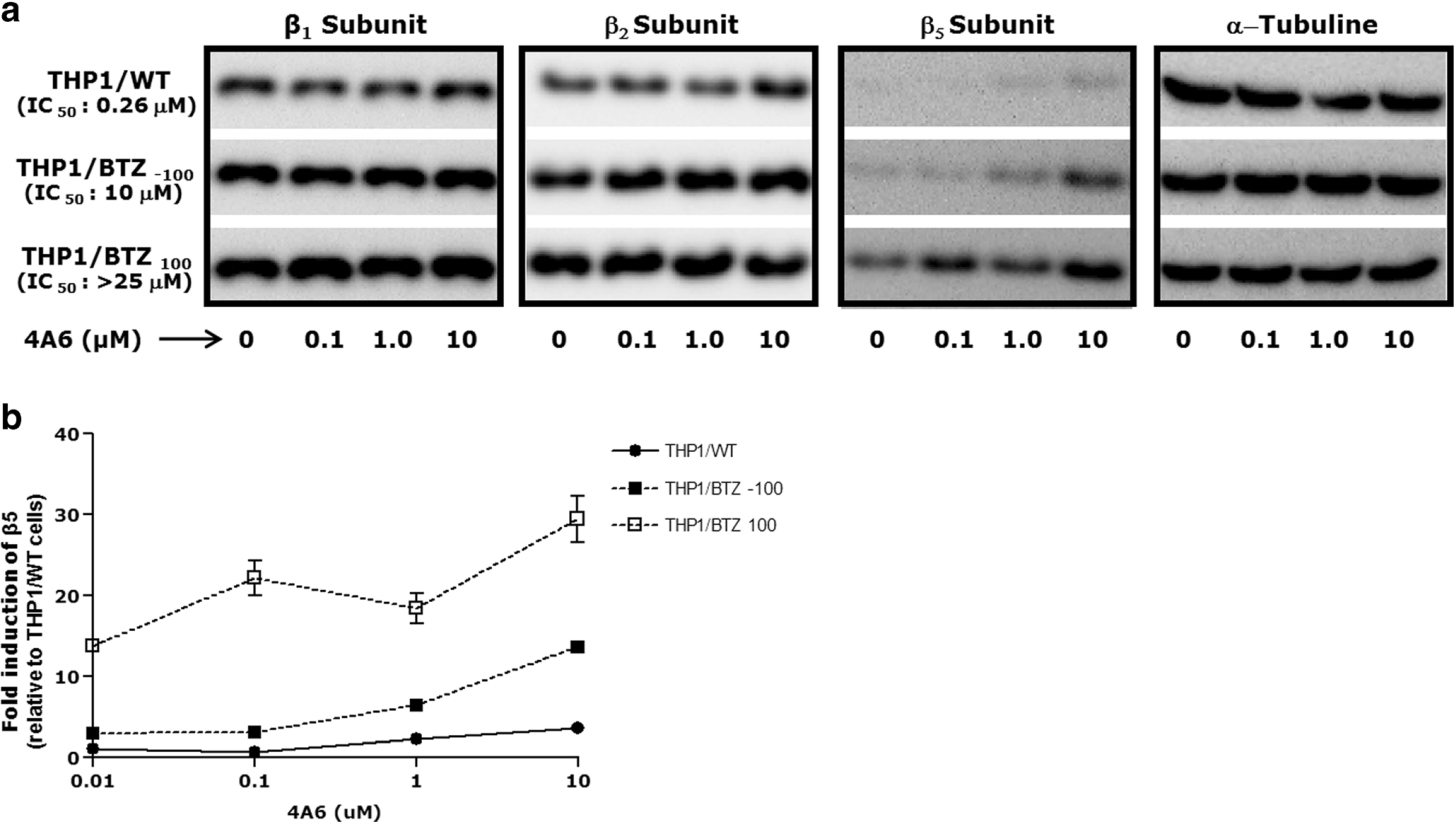
*4A6 induces proteasome β5 subunit overexpression.* **a** Protein expression of β1, β2 and β5 proteasome subunits in THP1/WT cells, the BTZ-resistant cell line THP1/BTZ_100_ grown in the presence of 100 nM BTZ, and THP1/BTZ _(−100)_ cells, a subline of THP1/BTZ_100_ that was grown in the absence of BTZ for 6 months. Before incubation with 4A6, THP1/BTZ_100_ cells were allowed a 4 days BTZ washout period (control/0), after which cells were exposed for 24 h to the indicated concentrations of 4A6. Expression of α-tubulin served as an actual loading control. **b** Results of scanning of protein band intensities in panel (**a**) are presented as mean ± S.D. of 3 separate experiments

Given the specific targeting of 4A6 of the β5 subunit of the proteasome, we explored whether exposure to 4A6 had an effect on the expression of the β5 subunit as compared to the other catalytic subunits β1 and β2. To this end, THP1/WT cells and the bortezomib-resistant cell lines THP1/BTZ _100_ and THP1/BTZ _(−100)_, the latter being a subline of THP1/BTZ _100_ that was grown in the absence of BTZ for 6 months, were exposed to a concentration range of 4A6 (0.1–10 μM) for 24 h (Fig. 7a). No significant effects of 4A6 exposure were observed regarding expression of the β1 and β2 proteasome subunits. In contrast, a dose-dependent increase in proteasome β5 subunit expression was noted in both THP1/WT sublines with relatively low basal levels of β5 expression and the two BTZ-resistant cell lines, including THP1/BTZ _(−100)_ cells that retained a level of cross-resistance to 4A6 similar as THP1/BTZ _100_ cells (Fig. 7a). Densitometric analysis showed a 3–5 fold increase in β5 subunit induction in all three cell lines upon exposure to 10 μM 4A6 exposure as compared to drug-free controls (Fig. 7b). This result implies that induction of proteasome β5 subunit expression constitutes a rapid adaptive response upon targeting of this subunit by the inhibitor 4A6.

## Discussion

Here we have shown that the cytotoxic hexameric 4A6 peptide elicits its pharmacological activity via selective and reversible inhibition of the chymotrypsin-like proteasome activity. The specific targeting of the chymotrypsin-like proteasome activity by 4A6 was further corroborated by upregulation of the expression of the β5 subunit of the proteasome. Moreover, cells harboring mutations in the β5 subunit which confer resistance to BTZ [21], displayed a marked cross-resistance to 4A6.

Most peptide-based proteasome inhibitors contain tri- or tetrapeptide moieties that dock into one or more of the active site pockets of the proteasome [16, 56]. However, peptides extended with N-terminally linked spacers and specific caps can also retain their proteasome inhibitory potential [50, 57]. Notwithstanding this fact, 4A6, as well as another hexameric peptide (4E11) exhibited a motif and mode of action distinct from known peptide-based proteasome inhibitors. The linear hydrophobic nature of 4A6 likely facilitates its interactions with the β5-subunit of the proteasome that preferentially cleaves after hydrophobic amino acid residues [12, 14]. To this end, we explored whether or not the interaction of 4A6 with the proteasome involves mere steric occlusion of the β5 active site or alternatively, that the 4A6 peptide serves as a cleavage substrate of the proteasome. Consistent with this notion may be the fact that the dimeric form of 4A6, which contains the same amino acid sequence as 4A6 and is therefore also likely to be cleaved by the proteasome, is almost equally effective in inhibiting β5-associated proteasome activity (Fig. 4a and Table 1). In this context, it is important to note that replacement of Thr(Bzl) at the P3 position by Lys(Z) or Ala in 4A6 abolished the cytotoxic effect by 4A6 [32], suggesting that this residue is essential for effective proteasome binding and inhibition.

The marked level of cross-resistance to 4A6 of cells resistant to the proteasome inhibitor BTZ (Table 1, Fig. 3) supports the conclusion that 4A6 and BTZ share a common mode of interaction with the β5 active site. In fact, studies from our laboratory revealed that the molecular basis of BTZ resistance in these cells involved a point mutation in the *PSMB5* gene that introduced a single amino acid change (Ala → Thr) at position 49 of the PSMB5 protein [46]. Since the Ala49 position resides in the BTZ binding pocket of PSMB5 and is involved in the interaction with BTZ [16, 30, 56, 58, 59], the Ala49Thr mutation is likely to underlie loss of BTZ binding and acquisition of bortezomib resistance [46]. The even higher levels of cross-resistance to 4A6 than resistance levels to BTZ suggest that Ala49 is even more critical in binding the 4A6 peptide than BTZ. In this respect, it was interesting to note that exposure of BTZ-resistant cells to 4A6 provoked a marked upregulation of mutant PSMB5 protein (Fig. 7), presumably as a compensatory mechanism to counteract loss of proteasome activity due to inhibition by 4A6.

Although the indicated PSMB5 mutation may be the dominant factor in conferring drug resistance to 4A6, it was previously reported that cellular extrusion by the MDR efflux transporters P-gp (ABCB1) and MRP1 (ABCC1) could also confer resistance to 4A6 [32]. This was further illustrated herein in the activity profile of 4A6 in the NCI60 panel of tumor cell lines where cells with a consistent MDR phenotype (mainly P-gp) were markedly less sensitive to 4A6 (Fig. 2). In contrast, such a MDR phenotype had relatively a marginal impact on the activity of BTZ. The presence of the boron group in BTZ most likely abolishes the ability of this compound to serve as a proficient substrate for MDR transporters as compared to other small peptides [60, 61]. Although Fig. 2 demonstrated an overlap in activities against some tumor types (leukemia/breast cancer), the current study indicates (Fig. 6d) that at least one mode of action of 4A6 was distinct from BTZ by not inducing apoptosis/growth inhibition via disregulation of mitochondrial calcium homeostasis [55]. Consistent with this study we showed here that inhibition of the mitochondrial uniporter with ruthenium red abrogated the growth inhibitory effects of BTZ, but had no effect on 4A6 activity.

Collectively, this study reported on 2 novel hexameric peptide-based proteasome inhibitors with several properties distinct from currently identified proteasome inhibitors, including BTZ. One of these peptides, 4A6, may serve as a lead compound for drug development by further optimization of its selective proteasome β5 subunit targeting against leukemia and breast cancer cells. The notion that 4A6 is a bona fide P-gp and MRP1 substrate may on the one hand compromise some of its activity against tumor cell expressing this drug efflux transporter, but on the other hand it may underlie a different, possibly more favorable toxicity profile than BTZ.

## Abbreviations

*BTZ*: bortezomib
*Reversin-P121*: Boc-Asp(OBzl)-Lys-(Z)-OtBu
*ALLN*: N-acetyl-Leu-Leu-norleucinal
*MG132*: Z-Leu-Leu-Leucinal
*MG262*: Z-Leu-Leu-Leu-boronate
*4A6*: Ac-Thr(tBu)-His(Bzl)-Thr(Bzl)-Nle-Glu(OtBu)-Gly-Bza
*4E11*: Ac-Thr(OBzl)-Glu(OtBu)-Glu(OBzl)-Asp(OtBu)-Glu(OtBu)-Gly-Bza
*Suc-LLVY-amc*: Suc-Leu-Leu-Val-Tyr-7-amino-4-methylcoumarin
*AAF-cmk*: Ala-Ala-Phe-chloromethylketone
*OtBu*: (O-)*tert*-butyl
*Bzl*: Benzyl
*Bza*: Benzylamine
*Ac*: Acetyl
*Ub*: Ubiquitin
*P-gp*: P-glycoprotein
*MRP1*: Multidrug resistance associated protein 1
*BCRP*: Breast cancer resistance protein
